# Rare cases of a second recurrence of nephroblastoma with MLLT1 gene mutation: case report and literature review

**DOI:** 10.3389/fonc.2024.1487544

**Published:** 2024-12-02

**Authors:** Yiling Dai, Xin Chen, Guoqian He, Ju Gao, Xia Guo

**Affiliations:** ^1^ Department of Pediatrics, West China Second University Hospital, Sichuan University, Chengdu, Sichuan, China; ^2^ Key Laboratory of Birth Defects and Related Diseases of Women and Children (Sichuan University), Ministry of Education, Chengdu, Sichuan, China; ^3^ Key Laboratory of Development and Diseases of Women and Children of Sichuan Province, West China Second University Hospital, Sichuan University, Chengdu, Sichuan, China; ^4^ West China Medical School of Sichuan University, Chengdu, Sichuan, China; ^5^ The Cardiac Development and Early Intervention Unit, West China Institute of Women and Children’s Health, West China Second University Hospital, Sichuan University, Chengdu, Sichuan, China

**Keywords:** nephroblastoma, Wilms tumor, recurrence, *MLLT1* gene, mutation

## Abstract

Nephroblastoma or Wilms tumor is the most common tumor of the urinary system in childhood. The survival rate can reach more than 90% after multidisciplinary treatment, but there is still a certain recurrence rate. In recent years, domestic and foreign scholars have analyzed the gene mutations related to the recurrence of nephroblastoma from the genetics or epigenetics perspective. However, few reports on the relationship between *MLLT1* and the pathogenesis have been reported; patients with *MLLT1* gene mutations are often associated with poor prognosis. In this case, we report the recurrence of nephroblastoma with *MLLT1* gene mutation and review relevant literature. The studies on molecular genetic mechanism will provide a theoretical basis for early warning, optimize individualized treatment plan, and are important for improving prognosis.

## 1 Introduction

Wilms tumor (WT), also named nephroblastoma, is the most common pediatric renal embryonal neoplasm. Its incidence rate ranks fourth in pediatric tumors, accounting for approximately 6% of children’s tumors and up to 90% of renal tumors in children (1). Metastasis occurs in the minority of patients, most frequently to the lung parenchyma, and to a lesser extent, the liver and regional lymph nodes (2). In recent decades, prognosis has improved secondary to surgical and medical advances and medical multidisciplinary group. The 5-year overall survival has improved to 90% (3). In patients without metastatic disease at presentation, approximately 75% of all recurrences occur within 1 year, and the relapse rate of patients enrolled in the combined International Society of Pediatric Oncology was 12%, with an overall survival in relapsed patients of 48% (4); thus, it is important to identify patients at risk of recurrence in order to adjust treatment in such a way that recurrence may potentially be prevented.

The exact pathogenesis underlying the recurrence of WT is still unclear, and the factors that influence the risk of recurrence include patient-, tumor-, and treatment-related characteristics. In recent years, biological markers, such as genetic alterations, have been studied more intensively, as these markers may be able to better identify patients at risk of tumor recurrence (5).

In addition to genetic changes identified within recurrent nephroblastoma (6), research has identified mutations that involve the highly conserved YEATS domain of MLLT1, a gene known to be involved in transcriptional elongation during early development. Children with MLLT1-mutant tumors present at a younger age and have a high prevalence of precursor intralobar nephrogenic rests (7). Through whole genome sequencing of pathological tissues from 82 recurrent nephroblastoma cases, the proportion of MLLT1 hotspot mutations was 6.1%, including two in the discovery set (n = 51) and three in the validation set (n = 31) (8). This is compared to a mutation rate of 19/533 (3.6%) among newly diagnosed nephroblastoma.

We report two cases of a second recurrence of nephroblastoma with *MLLT1* mutation. It could provide evidence that supports the role of *MLLT1* mutation in the pathogenesis of a subset of nephroblastoma. It might provide a theoretical basis for predicting the risk of recurrence.

## 2 Case series

### 2.1 Case 1

#### 2.1.1 Clinical manifestation

A 6-month-old male infant was admitted to West China Second University Hospital and presented with an abdominal mass. He had no fever, vomiting, abdominal pain, or bloating and no family history of malignant tumors. A lump (approximately 6.0 cm × 8.0 cm) was palpable in left down abdomen, with unclear boundaries, poor mobility, and without tenderness. Abdominal computed tomography showed a huge abnormal soft tissue mass (approximately 7.3 cm × 6.7 cm) in the left kidney, with patchy high-density shadows. Irregular enhancement appeared in the circumference or parenchyma of tumors in enhanced CT scan sequences.

#### 2.1.2 Treatment

**Figure 1:**
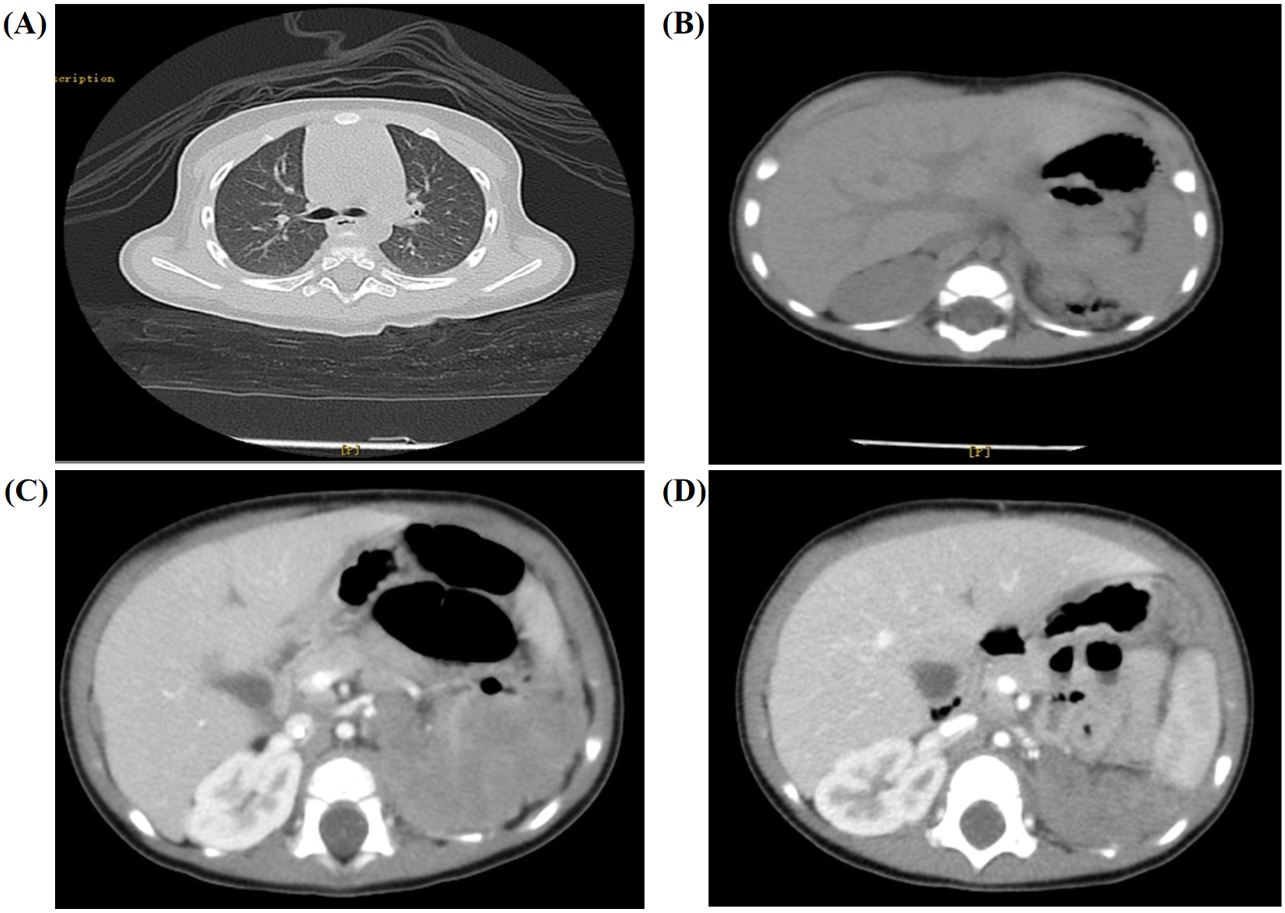
Imaging results for the 6-month-old male infant. **(A, B)** Chest and abdominal CT showed no residual tumor or distant metastasis. **(C)** After completing the 6 weeks of chemotherapy, abdominal CT showed irregular soft tissue mass in the left retroperitoneum (approximately 6.3 cm × 3.8 cm × 6.7 cm). **(D)** Abdominal imaging indicated that the irregular soft tissue mass gradually shrunk after a new round of chemotherapy with the protocol CCCG-WT-2019 (Regime I).

**Figure 2:**
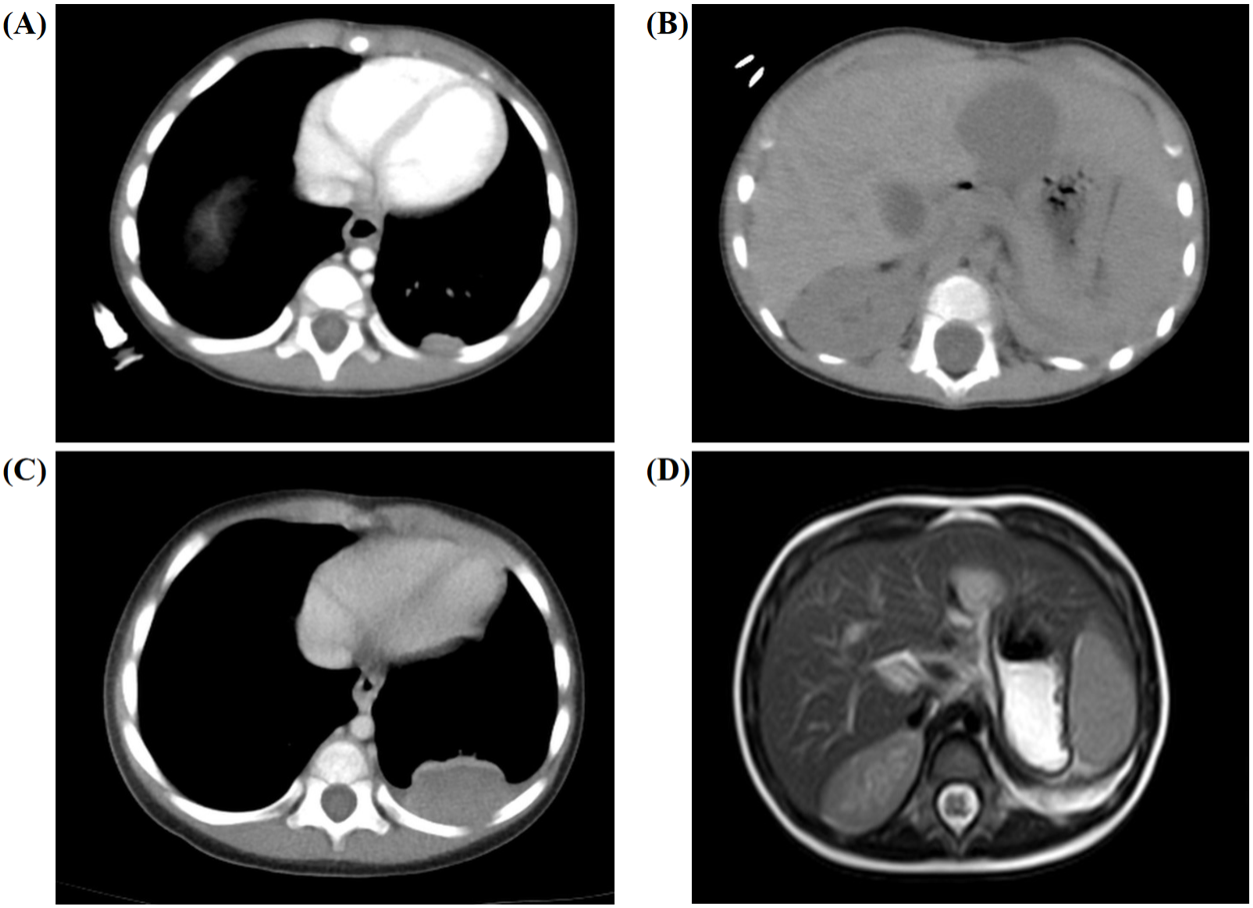
Secondary chemotherapy and postoperative imaging results for the 6-month-old male infant. **(A, B)** Abdominal CT showed new space-occupying lesions in the liver (approximately 2.9 cm × 3.0 cm × 2.7 cm) and lung (approximately 1.2 cm × 0.5 cm) after completing 16 weeks of chemotherapy. **(C, D)** Chest CT and abdominal MRI showed no relief of the multiple new masses and nodules during the whole treatment.

The patient received laparoscopic left renal and retroperitoneal giant tumor resection at another hospital on the 7th day after the discovery of the abdominal mass. The tumor (approximately 8.0 cm × 7.0 cm × 6.0 cm) originated from the left kidney and had extensive adhesions with surrounding tissues. Due to the intact capsule, it could be completely removed. The pathology and immunohistochemistry confirmed the diagnosis of nephroblastoma (favorable histology, mixed). Chest and abdominal CT (**Figures 1A, B**) showed no residual tumor or distant metastasis. According to the recommendations for the diagnosis and treatment of nephroblastoma issued by the Chinese Children Cancer Group in 2019 (CCCG-WT-2019), the tumor was classified as stage 1: (1) the tumor was limited to the kidneys and was completely removed surgically; (2) the renal capsule was intact; (3) there was no rupture before tumor resection; (4) there was no invasion in the renal sinus; (5) there was no residual tumor at the surgical margin; and (6) all sampled lymph nodes were negative. On the 24th day after the first surgery, the patient received chemotherapy with the protocol CCCG-WT-2019 (Regime EE4A). This regimen included vincristine and dactinomycin, with a total course of 19 weeks. However, after completing 6 weeks of chemotherapy, abdominal CT (**Figure 1C**) showed irregular soft tissue mass in the left retroperitoneum, which was suspected to be early tumor recurrence. The patient underwent a second operation for retroperitoneal tumor resection and biopsy, and the pathology supported the recurrence of Wilms tumor. On the 23rd day after the second surgery, he started a new round of chemotherapy with the protocol CCCG-WT-2019 (Regime I). This regimen included vincristine, adriamycin, etoposide, and cyclophosphamide, with a total course of 24 weeks. During the treatment, abdominal imaging (**Figure 1D**) indicated that the irregular soft tissue mass gradually shrunk. The treatment appeared to be progressing in a positive direction. Nevertheless, after completing 16 weeks of chemotherapy, abdominal CT (**Figures 2A, B**) showed new space-occupying lesions in the liver and lung, which were suspected to be disease progression. Subsequently, we adjusted the chemotherapy regimen to the protocol VIT, which included vincristine, irinotecan, and temozolomide. However, during this treatment, chest CT and abdominal MRI (**Figures 2C, D**) showed no relief of multiple new masses and nodules. Eventually, his parents discontinued the chemotherapy, and the child died from disease progression.

#### 2.1.3 Identification and characterization of MLLT1 variants

**Figure 3:**
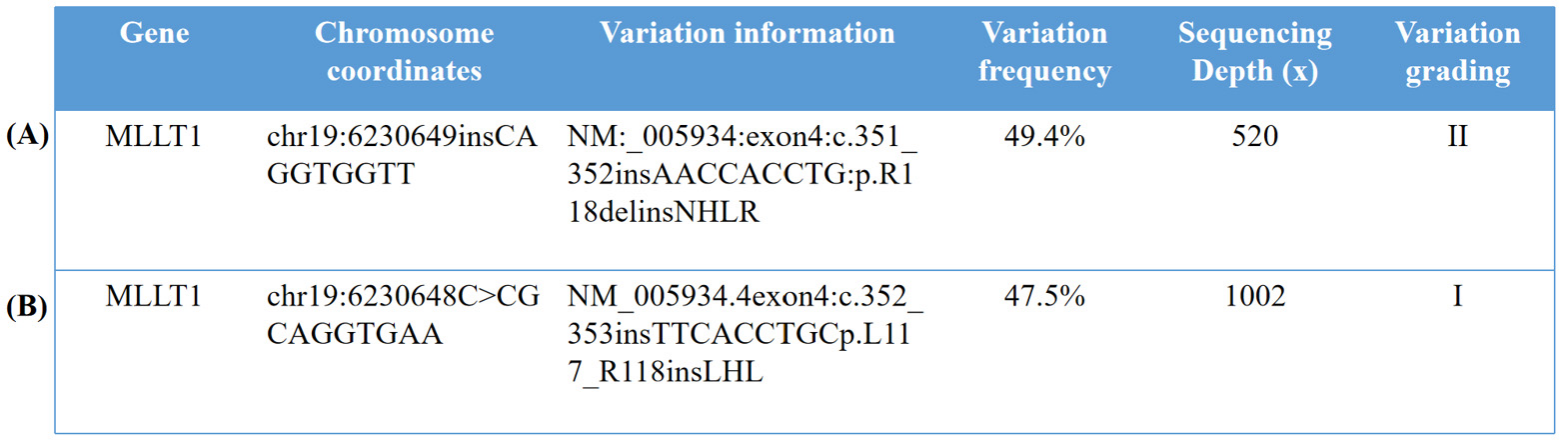
Identification and characterization of MLLT1 variants. **(A)** The whole genome chip detection of the solid tumor sample of the 6-month-old male infant showed no loss of heterozygosity (LOH) variation in the 1p and 16q regions, no repetitive variation in the 1q region, and no other acquired chromosome copy number variation (CNV) changes. **(B)** The results of the whole genome chip detection sample of the 9-month-old male infant were 0 acquired CNV abnormalities, no LOH variation found in the 1p region and the 16q region, and no acquired variation was found in the 1q region. (Variation grading in the table is divided into grade I, variation clearly related to the disease; level II, variations that may be associated with the disease; and grade III, variants with unknown clinical significance.). 1. In our study, the reference genome of the chromosome location was GHCh37/hg19. 2. In our study, the detection method used for the two children with nephroblastoma was whole-genome sequencing. The whole genome chip belongs to Illumina cytoSNP-850K chip. The basic principle is to connect the probe to the microbeads, and then, the microbeads carrying the probe are randomly adhered to the chip. The DNA of the sample to be tested is hybridized with the probe and the single base extension is performed. The copy number variation (CNV) of the sample to be tested is analyzed by scanning the fluorescence signal. The probe position can cover the whole genome of 22 pairs of autosomes and sex chromosomes and can detect chromosome copy number abnormalities (deletion or duplication) and uniparental disomy (UPD) with a normal copy number.

The tumor gene analysis (**Figure 3A**) detected nine nucleotide in-frame insertions (AACCACCTG) at *MLLT1* position c.351_352 (p.R118delinsNHLR) of the NM_005934, with a mutation frequency of 49.40%. No loss of heterozygosity (LOH) mutation was found in the 1p and 16q regions, and no repetitive mutation was found in the 1q region.

### 2.2 Case 2

#### 2.2.1 Clinical manifestation

A 9-month-old male infant started with abdominal distension and abdominal mass without a family history of malignant tumors. Ultrasound showed a medium echo mass (approximately 7.8 cm × 6.5 cm × 6.9 cm) in the right kidney, with a clear boundary and regular shape. There was a dark patchy liquid area, and dotted and linear blood flow signals were seen inside and around the sound transmission difference mass.

#### 2.2.2 Treatment

**Figure 4:**
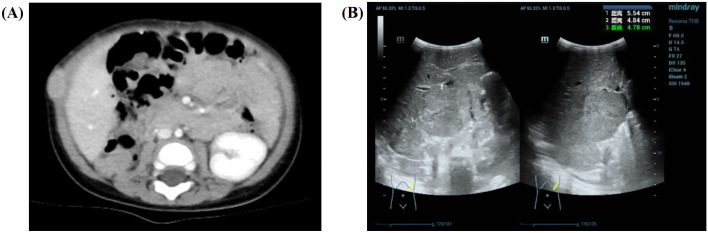
Imaging results for the 9-month-old male infant. **(A)** The soft tissue density nodules (approximately 2.1 cm × 1.1 cm × 2.2 cm) in the posterior part of the operation area are seen in the postoperative abdominal CT after surgery. **(B)** Ultrasound reexamination at the end of the 6th week of chemotherapy showed that the multiple lesions in the liver (approximately 5.5 cm × 4.8 cm × 4.8 cm) and abdominal cavity were not relieved.

Approximately 10 days after the discovery of the abdominal mass, the patient underwent radical resection of a huge space-occupying lesion in the right kidney, and the tumor was completely removed during the operation. In the process of operation, a small amount of bloody ascites was found in the abdominal cavity, the volume of the right kidney was enlarged, and a huge mass in the upper and middle parts could be found (8.0 cm × 6.0 cm × 5.0 cm). There was an enlarged lymph node near the right abdominal aorta. Final pathology and immunohistochemistry confirmed the diagnosis of Wilms tumor (favorable histology, mixed). Since the patient underwent the first complete resection of the abdominal mass in another hospital, no radiotherapy and chemotherapy were performed after the evaluation. Therefore, the child's imaging data were not available before the surgery. Forty-eight days after the operation, the child’s parents noticed a new mass near the incision, and they went to our hospital 55 days after the operation. Although no residual tumor or distant metastasis was found in the patient’s chest CT for the first time after the operation, soft tissue density nodules in the posterior part of the operation area were seen in the postoperative abdominal CT (**Figure 4A**); the boundary between the adjacent peritoneum and the local intestinal wall was not clear; and the nodular thickening of the perihepatic peritoneum and the soft tissue density nodules in the right upper abdominal wall suggested the possibility of tumor recurrence and metastasis. According to the recommendations for the diagnosis and treatment of nephroblastoma issued by the Chinese Children Cancer Group in 2019 (CCCG-WT-2019), the tumor was classified as stage 3: for tumor metastasis of the right upper abdominal wall and lymph node involvement of the abdominal and pelvic cavity. With the influence of different medical institutions and the family’s social standing, the patient was able to undergo chemotherapy with the protocol CCCG-WT-2019 (Regime DD4A) on the 60th day after surgery, which was assessed 5 days after confinement in our hospital. This regimen included vincristine, adriamycin, and dactinomycin, for a total course of 25 weeks. However, after 3 weeks of chemotherapy, the patient’s abdominal CT showed multiple nodules and masses in the abdominal cavity and retroperitoneal space, suggesting multiple abdominal metastases. His ultrasound reexamination (**Figure 4B**) at the end of the 6th week of chemotherapy showed that the multiple lesions in the liver and abdominal cavity were not relieved. Finally, the child’s parents discontinued the treatment after careful consideration.

#### 2.2.3 Identification and characterization of MLLT1 variants

The tumor gene analysis (**Figure 3B**) detected nine nucleotide in-frame insertions (TTCACCT GC) at MLLT1 position c.352_353 (p.L117_R118insLHL) of the NM_005934.4 with a mutation frequency of 47.50%. No 1p LOH, 1q gain, 11p15 LOH, 16q LOH, and 17p13.1 loss were detected in this patient’s pediatric nephroblastoma genome-wide chip.

## 3 Discussion

The development of tumors is a multifactorial and multi-stage process, which is the result of the comprehensive effects of host, genetic, and environmental factors. Nephroblastoma is an embryonal cancer and is thought to be derived from nephrogenic rests (NRs), which are the foci of embryonic kidney precursor cells (9). In the past two decades, an increasing number of findings emerging from genetic and cytogenetic studies have improved our ability to characterize changes in genes involved in WT, which include *WT1*, *CTNNB1*, *IGF2*, and *WTX* (10, 11).

Recently, a series of hot spot mutations in the YEATS domain of ENL, also known as MLLT1, have been identified in 5%–9% of nephroblastoma patients, making it the most frequently mutated epigenetic regulator in the disease (12). *MLLT1* gene is a newly discovered gene likely associated with the pathogenesis of nephroblastoma. It is located on chromosome 19p13.3 and encodes a 559aa protein from 12 exons. In the human study, ENL (MLLT1, YEATS1) and AF9 (MLLT3, YEATS3) are the most studied and highly homologous. The >80% homology between AF9 and MLLT1 domains supports the similar function of MLLT1. MLLT1 protein is a key component of the hyperelongation complex, regulating mRNA elongation through RNA polymerase II (RNAPII) during transcription to regulate RNA expression, while perturbation of transcription elongation has been shown to cause abnormal development and neoplastic transformation. The N-terminal region of MLLT1 protein contains a YEATS domain, which can function as a reader protein, specifically recognizing and binding H3K9ac, bridging the chromatin template with DOT1L, resulting in H3K79 trimethylation, and hence, activating the transcription of associated genes (13, 14). Important residue differences between MLLT1 and AF9 involve 107 and 111 positions within loop 8 (L8), which plays an important role in regulating the affinity and specificity of the YEATS domain. On the one hand, the binding affinity of AF9 YEATS domain H3K9 ac is higher than that of ENL (15). The mutant MLLT1 YEATS domain showed substantially altered affinity with H3K9ac, thereby affecting the transcriptional regulation of early kidney development. Allis et al. studied the effects of these mutations on transcription. These mutations accumulate in the YEATS domain and do not redistribute ENL to new target sites to a large extent. On the contrary, each mutant showed enhanced occupancy on the ENL target gene subset, which was closely related to gene activation. MLLT1 (ENL) mutation can drive nephroblastoma by enhancing the phase separation and transcription of target genes (16). In addition, the dysregulation of several *HOX* genes and upregulation of MYC in MLLT1-mutant FHWT provide indirect evidence for an altered transcriptional regulation in MLLT1-mutant tumors, as these genes are recognized to be regulated through transcriptional elongation via the super-elongation complex (17, 18). MLLT1 mutations in nephroblastoma can also mediate HOX disorders. The evidence is clearest in the significant and early overexpression of HOXA13. Of particular interest was the high expression of *HOXA13*, a gene not expressed in the FHWT or the cap mesenchyme of the developing kidney (19). *HOXA13* upregulation has been shown to be a poor outcome marker in some adult cancers, but the role of *HOXA13* overexpression in WT development is not apparent. The association between *MLLT1* mutation and *HOXA13* overexpression is supported by the accompanying increased expression of HOTTIP, an lncRNA that can result in transcriptional activation of *HOXA13* (20).

Apart from affecting the transcriptional regulation, the *MLLT1*-mutant tumors were also accompanied by *CTNNB1* or *WTX* mutations, which would activate the Wnt pathway, resulting in increased proliferation during early development (21). The abnormal proliferation of early undifferentiated cells could remain localized, or as the early kidney grows, they could separate into multiple aggregates, resulting in the subsequent development of single or multiple intralobar nephrogenic rests and WT may develop within them. Hence, it may be speculated that the patients carrying *MLLT1* mutations may present at a younger age, are more likely to develop multi-foci multiple intralobar nephrogenic rests, and have a higher risk of nephroblastoma.

Here, we reported the in-frame insertion *MLLT1* mutation (NM_005934: exon4:c.351_352ins and NM_005934.4:exon4:c.352_353ins) in favorable histology nephroblastoma. The identical *MLLT1* insertion variant was not identified in other tumors (neuroblastoma, lymphoma, leukemia, and osteosarcoma), and copy number loss at chromosome 19p13 was not identified. Similar to literature reports, the onset age is <1 year old, consistent with the characteristics of young onset age. This suggests that this specific cellular environment may be limited to early embryonic kidneys (22, 23). A team speculated that in patients with tumors carrying MLLT1 mutations, these mutations occur in undifferentiated cells in the early stage of renal development. It further illustrates that WT with MLLT1 mutations presents at a younger age (7). Despite early and aggressive treatment, the patients experienced a second recurrence. Although no detectable correlation between the specific *MLLT1* mutation and relapse was clarified, we highly suspect that the *MLLT1* mutation status is likely to increase the risk of recurrence. We did not detect intralobar nephrogenic rests and identify any evidence of germ-line mutations; therefore, we were not able to clarify the relationship between *MLLT1*-mutant tumors and intralobar nephrogenic rests.

## 4 Conclusion

In summary, we report a somatic mutation in *MLLT1* and provide evidence that patients with *MLLT1*-mutant tumors present at a younger age. Further understanding the function of *MLLT1* mutations in nephroblastoma could provide significant insight into the development of nephrogenic rests and risk factors for tumor relapse.

## Data Availability

The raw data supporting the conclusions of this article will be made available by the authors, without undue reservation.
